# Posterior cruciate ligament reconstruction using PCL inlay technique with the patient supine in bicruciate ligament injury reconstruction

**DOI:** 10.1186/s13018-022-03495-6

**Published:** 2023-01-06

**Authors:** Sergio Rocha Piedade, Gerson Muraro Laurito, Filippo Migliorini, Nicola Maffulli

**Affiliations:** 1 Exercise and Sports Medicine, Department of Orthopedic, Rheumatology, and Traumatology, University of Campinas - UNICAMP, School of Medical Sciences, Campinas, SP Brazil; 2 Department of Orthopaedic, Trauma, and Reconstructive Surgery, RWTH University Hospital of Aachen, 52074 Aachen, Germany; 3 Department of Musculoskeletal Disorders, School Medicine, Surgery and Dentistry, University of Salerno, Pauwelsstraße 30, 84081 Baronissi, Italy; 4 Centre for Sports and Exercise Medicine, Barts and The London School of Medicine and Dentistry, Mile End Hospital, Queen Mary University of London, 275 Bancroft Road, London, E1 4DG England, UK; 5 School of Pharmacy and Bioengineering, Keele University School of Medicine, Thornburrow Drive, Stoke on Trent, England, UK

**Keywords:** Posterior cruciate ligament injuries, Posterior cruciate ligament reconstruction, Surgical procedures, operative, Surgical instruments

## Abstract

**Background:**

Surgical reconstruction of the posterior cruciate ligament (PCL) can be technically challenging given the proximity of the popliteal artery to the PCL tibial insertion. This "no-safe zone" makes some knee surgeons less confident and willing to perform this surgical procedure.

**Surgical technique:**

We present a PCL tibial inlay reconstruction technique using a set of instruments involving three tools (a slot cut, a bone plug positioner, and an impactor).

**Conclusion:**

This set of instruments allows a more reproducible posteromedial approach and to produce a PCL tibial slot in a posterior cruciate ligament inlay reconstruction with the patient supine in bicruciate ligament injury reconstruction.

## Introduction

**Fig. 1:**
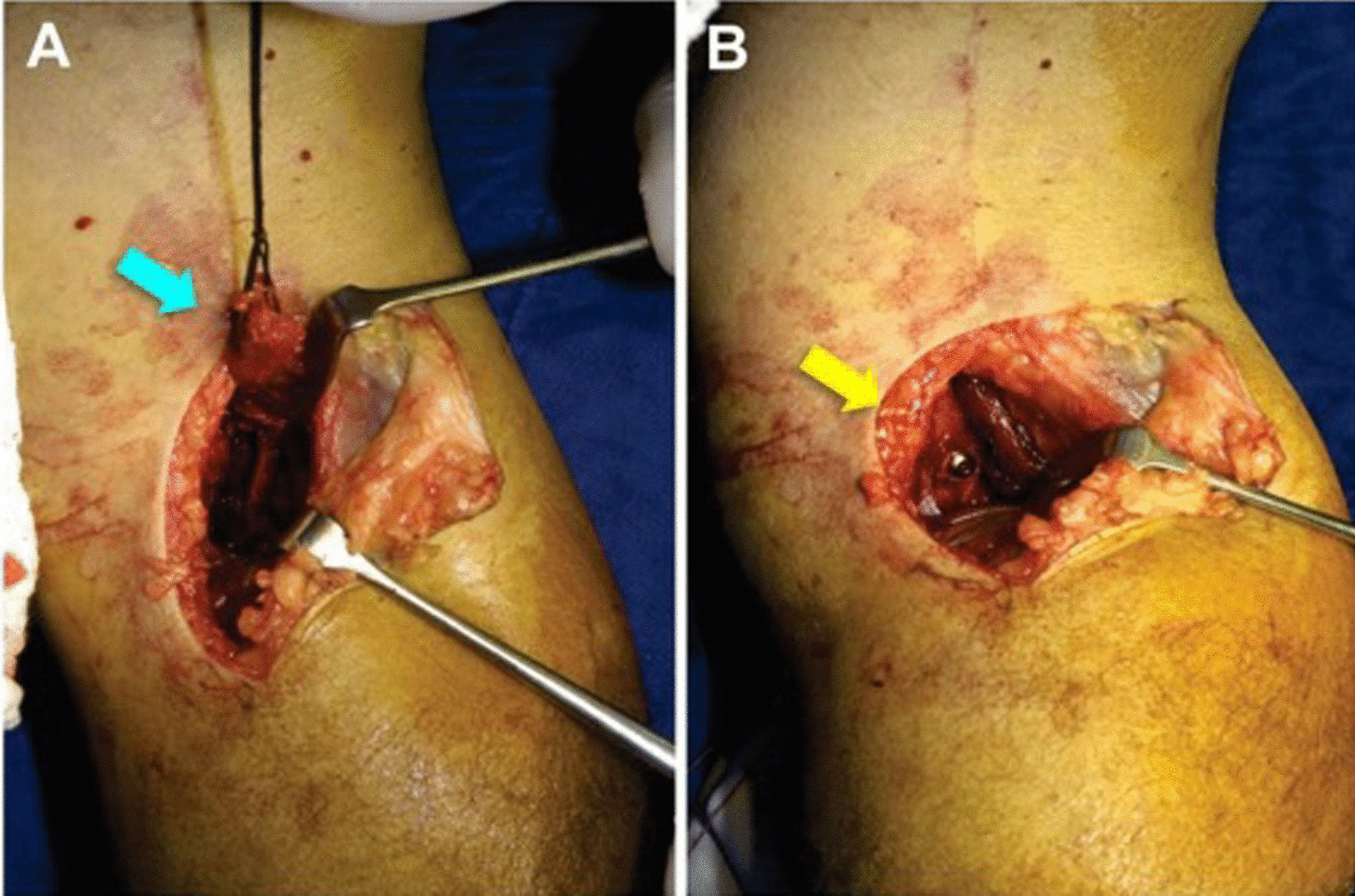
Posteromedial approach with the patient prone: **A** the bone block of the bone-patellar tendon-bone graft (blue cyan arrow); **B** after fixation of the bone block at the insertion of the PCL on the tibia (white arrow)

Surgical reconstruction of the posterior cruciate ligament (PCL) can be technically challenging given the proximity of the popliteal artery to the PCL tibial insertion [1, 2]. This area, the “no-safe zone”, makes some knee surgeons less confident and willing to perform this surgical procedure [3–5]. Surgical reconstruction of the PCL can be performed using two main techniques: the tibial tunnel or the tibial inlay technique [6, 7]. The tibial tunnel technique approaches the PCL tibial insertion using a specific guide under arthroscopic control and, in some circumstances, fluoroscopic control. The PCL tibial inlay technique allows direct approach to the tibial insertion of the PCL [8–10]. Both methods seem to produce equivalent results [1, 2, 11]. Since 2002, we have used the PCL inlay open technique, positioning the patient prone to undertake a posteromedial expose the tibial insertion of the PCL (Fig. 1) [5, 13, 14].

**Fig. 2:**
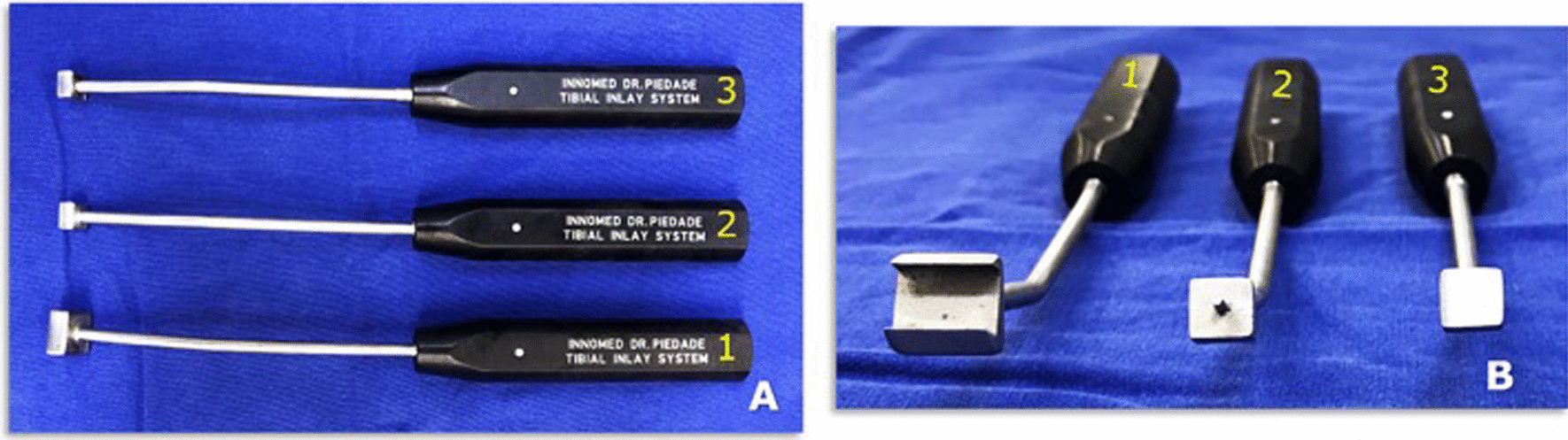
Lateral (**A**) and front (**B**) views of the Piedade tibial Inlay set of instruments: slot cut (osteotome), (2) positioner, and (3) impactor

We originally, used an open technique [5, 15, 16]. Since 2009, drilling of the PCL femoral tunnel has been performed arthroscopically, and the tibial insertion of the PCL has been approached in an open fashion with the patient supine [17, 18]. We developed a PCL tibial inlay system for a more reproducible posteromedial approach and bone plug positioning with the patient supine. This set of instruments involves three tools (a slot cut, a bone plug positioner, and an impactor) that allow to reproducibly produce a PCL tibial slot for bone plugs of placement of the PCL tendon-bone graft Fig. 2.

## Indication

Non-surgical management is normally indicated for grade I and II PCL injuries, but the surgery can be considered for grade II posterior instability. Surgery is considered mandatory for grade III (15-mm posterior knee displacement) PCL tears, and complex knee ligament injuries, including bicruciate injury, and PCL tears associated with peripheral components [6–8]. Obviously, decision-making should be based on thorough clinical assessment and stress radiographs (https://pubmed.ncbi.nlm.nih.gov/19464187/).

## Surgical technique–no changing of patient decubitus

1. With the patient supine and under spinal anaesthesia, the knee is kept at 90° flexion with a support on the lateral aspect of the proximal 1/3 of the thigh and another under the foot. After exsanguination, the tourniquet is inflated to 300 mmHg.
2. A routine diagnostic arthroscopy of the knee is performed to confirm the diagnosis. If necessary, meniscal injuries are addressed.
3. The PCL and anterior cruciate ligament (ACL) stumps are resected.
4. The PCL and ACL femoral tunnels are drilled using an outside-in PCL guide positioned at the origin of the PCL and ACL in the femur, under arthroscopic control, respectively (Fig. 3A and B). Initially, the bone tunnel has a 6 mm diameter and will be adjusted later, according to the diameter of the harvested graft.

**Fig. 3:**
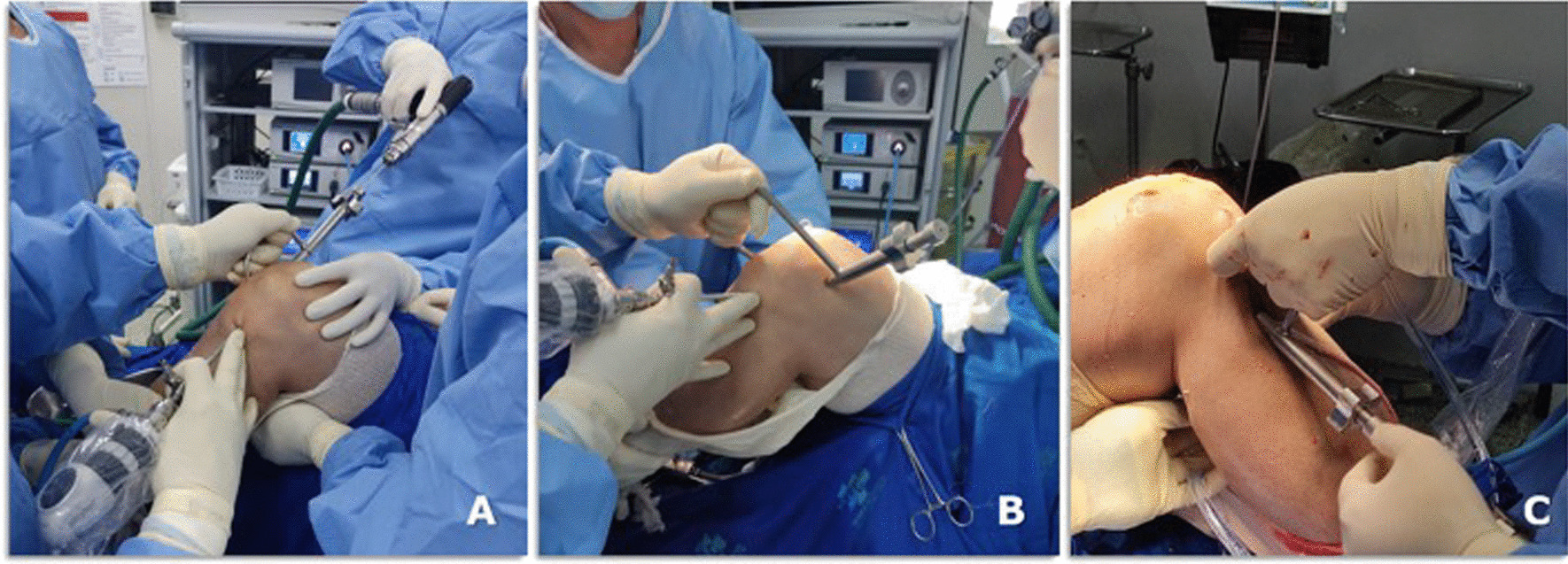
Femoral tunnel positioning of PCL (**A**) and (**B**) ACL (**B**) tunnels, and (**C**) ACL tibial tunnel under arthroscopic control
5. Then, the central third of the quadriceps tendon with patella bone plug is harvested through a midline longitudinal 7-cm incision
6. Through a 5-cm longitudinal incision on the pes anserinus, the gracilis and semitendinosus tendons are harvested with an open stripper, keeping them attached to their insertion on the tibia
7. The diameter of the PCL and ACL femoral tunnels is adjusted after measuring the diameter of the final graft.
8. Using the incision used to harvest the hamstring tendons, the ACL tibial tunnel is drilled at a 55° angle using a tibial guide under direct arthroscopic control (Fig. 3C)
9. After all bone tunnels have been drilled, the length of ACL graft (double gracilis-semitendinosus tendon graft) is measured and the graft prepared using vicryl 2.0

## Knee posteromedial approach with the patient supine

10. The hip is kept in external rotation and the knee at 90 degrees of flexion (Fig. 4A and B)

**Fig. 4:**
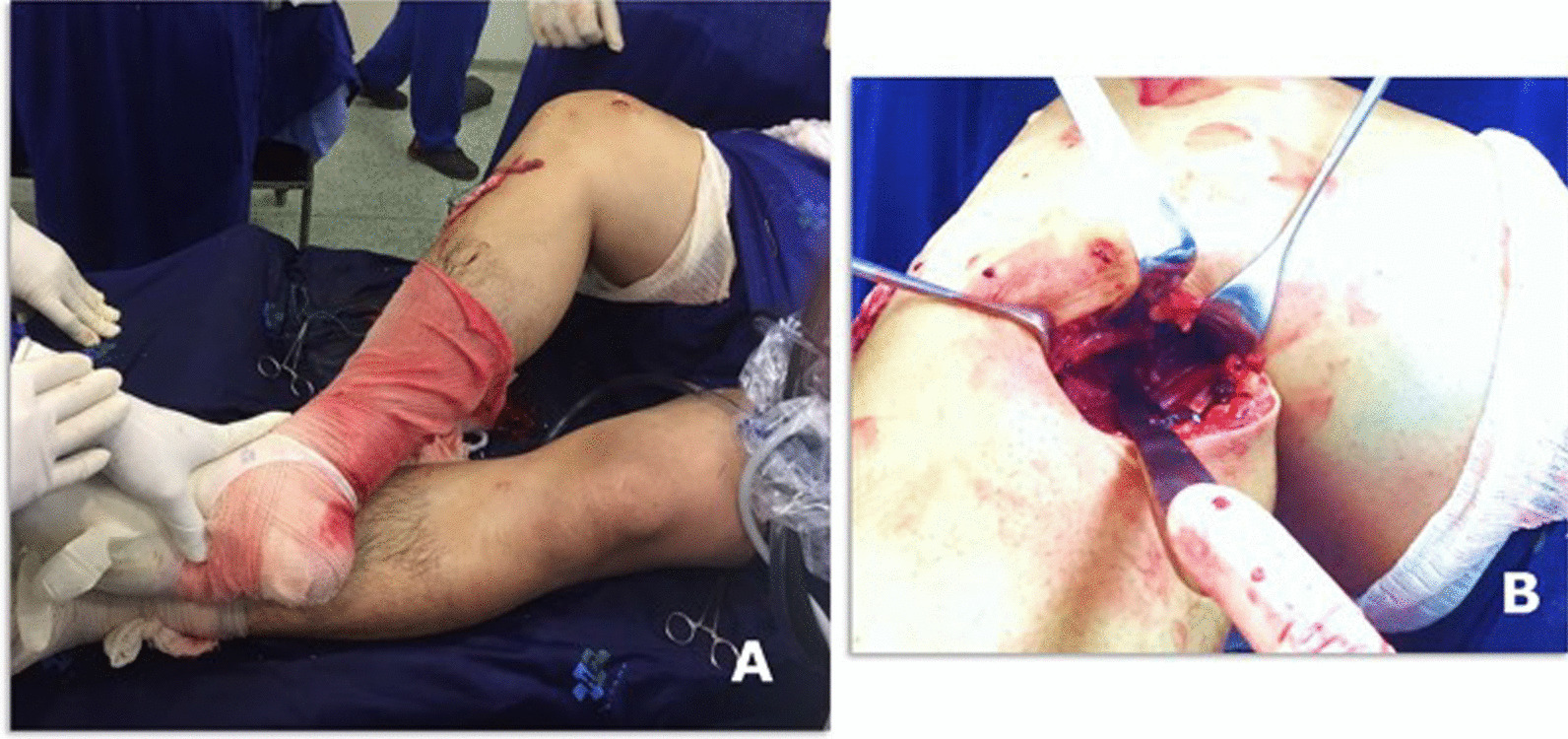
Knee posteromedial approach: Lower limb positioning with external hip rotation, and the knee flexion (**A**) and intraoperative view of posteromedial approach of PCL tibial insertion in the right knee (**B**)
11. An a L-inverted posteromedial incision curved incision is performed, with the horizontal branch of the L places in the knee flexion crease (Fig. 5A)

**Fig. 5:**
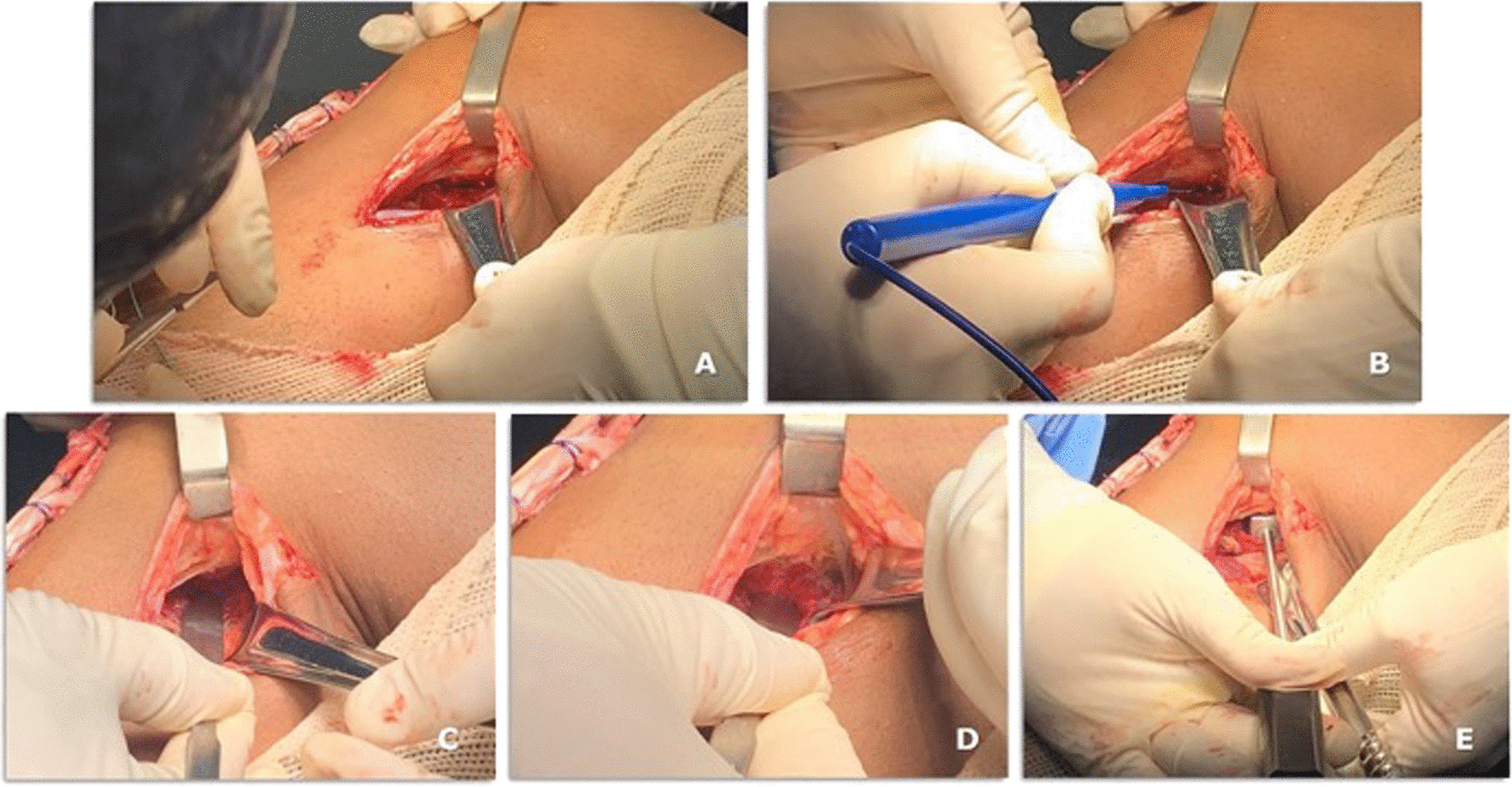
A case of bicruciate ligament reconstruction showing the posteromedial approach (**A**–**D**) and the Piedade PCL tibial INLAY bone slot cut is positioned on the PCL tibial insertion (**E**)
12. The deep fascia is incised vertically (Fig. 5B)
13. After blunt dissection between the medial border of the gastrocnemius muscle and semimembranosus tendon, the medial border of the gastrocnemius is retracted laterally and posteriorly to expose the posterior capsule (Fig. 5C–E).
14. The capsule is opened using a medial vertical incision, and the PCL tibial insertion is visualized Fig. 5D
15. Then, the Piedade PCL tibial inlay bone slot cut is positioned on the PCL tibial insertion, and a hammer is used to tap and produce a bone slot (10 mm long, 9 mm wide, and 10 mm deep) (Figs. 5E, 6A–C and, 7A).
16. After removing the cortical bone of this slot (Fig. 6D), the Piedade PCL tibial inlay bone slot impactor is used to regularize the walls of the slot and its depth according to the dimensions of the harvested patella bone plug (Figs. 6E and 7B).

**Fig. 6:**
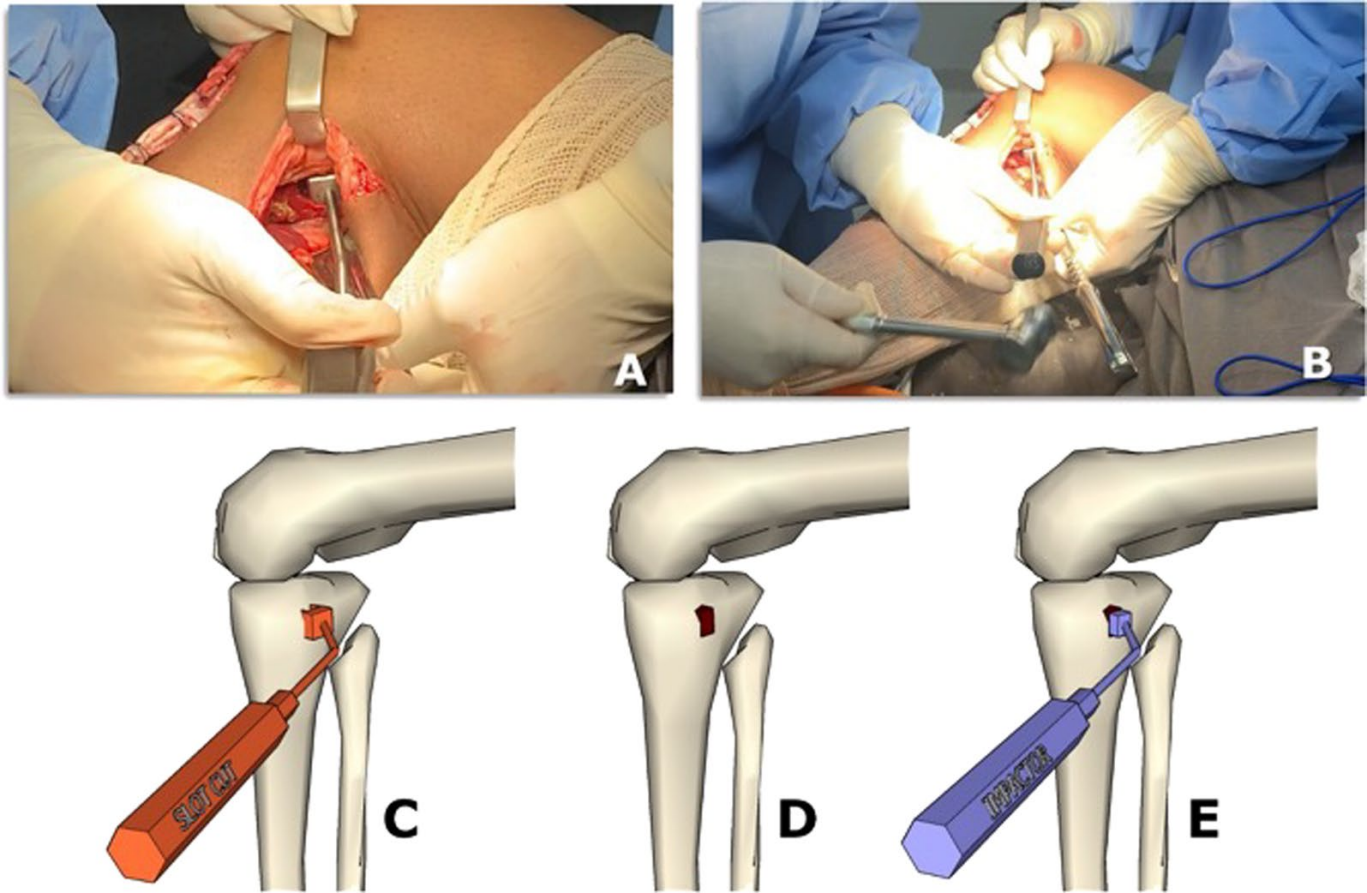
Producing the PCL tibial slot using the set of instruments: slot cut (**A** and **B**) and impactor (**C** and **D**) to control the depth of the slot according to the graft bone block. Note that the lower leg is externally rotated during this phase of the procedure

**Fig. 7:**
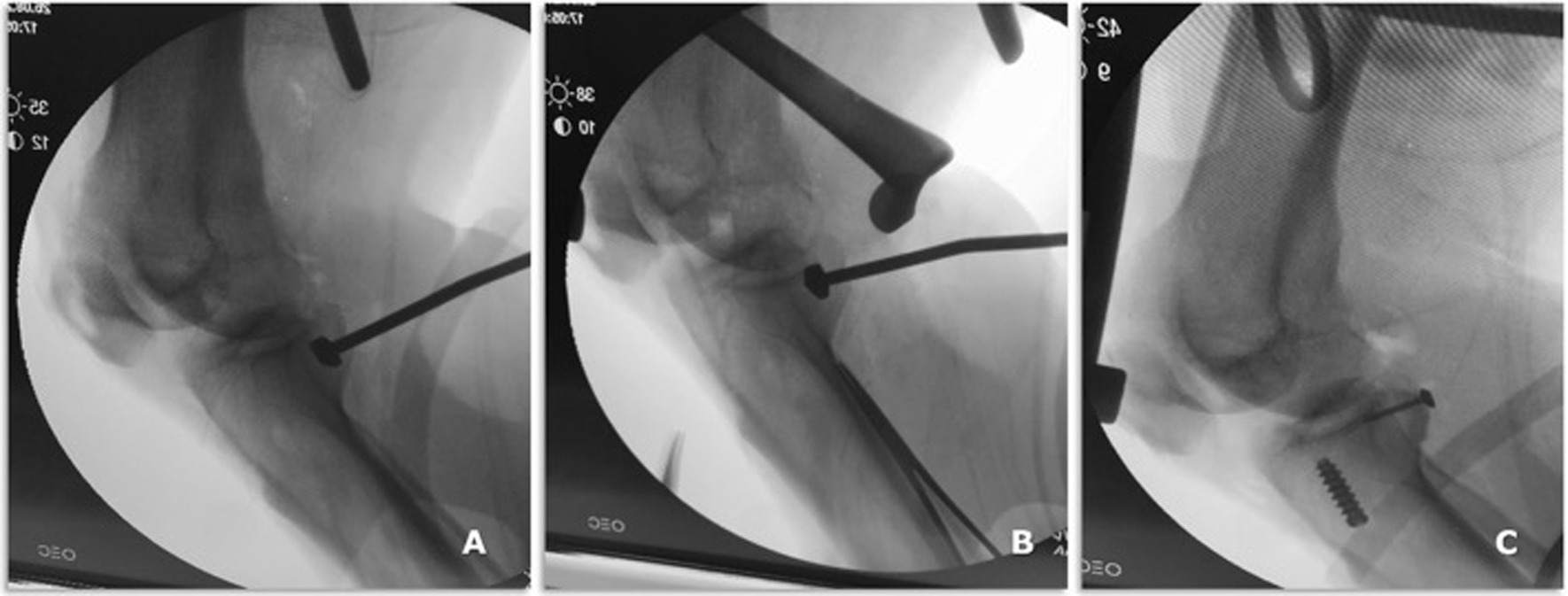
Intraoperative radiographic view of osteotome (**A**) and impactor (**B**) positioning on the PCL tibial bed and PCL and ACL graft fixation on the tibial side (**C**)
17. Before passing the graft, a pull-out bone tunnel is added to the graft fixation by drilling a 2.5-mm tunnel just below the PCL bone slot from the posterior to the anterior aspect of the knee joint (Figs. 8A, 9)

**Fig. 8:**
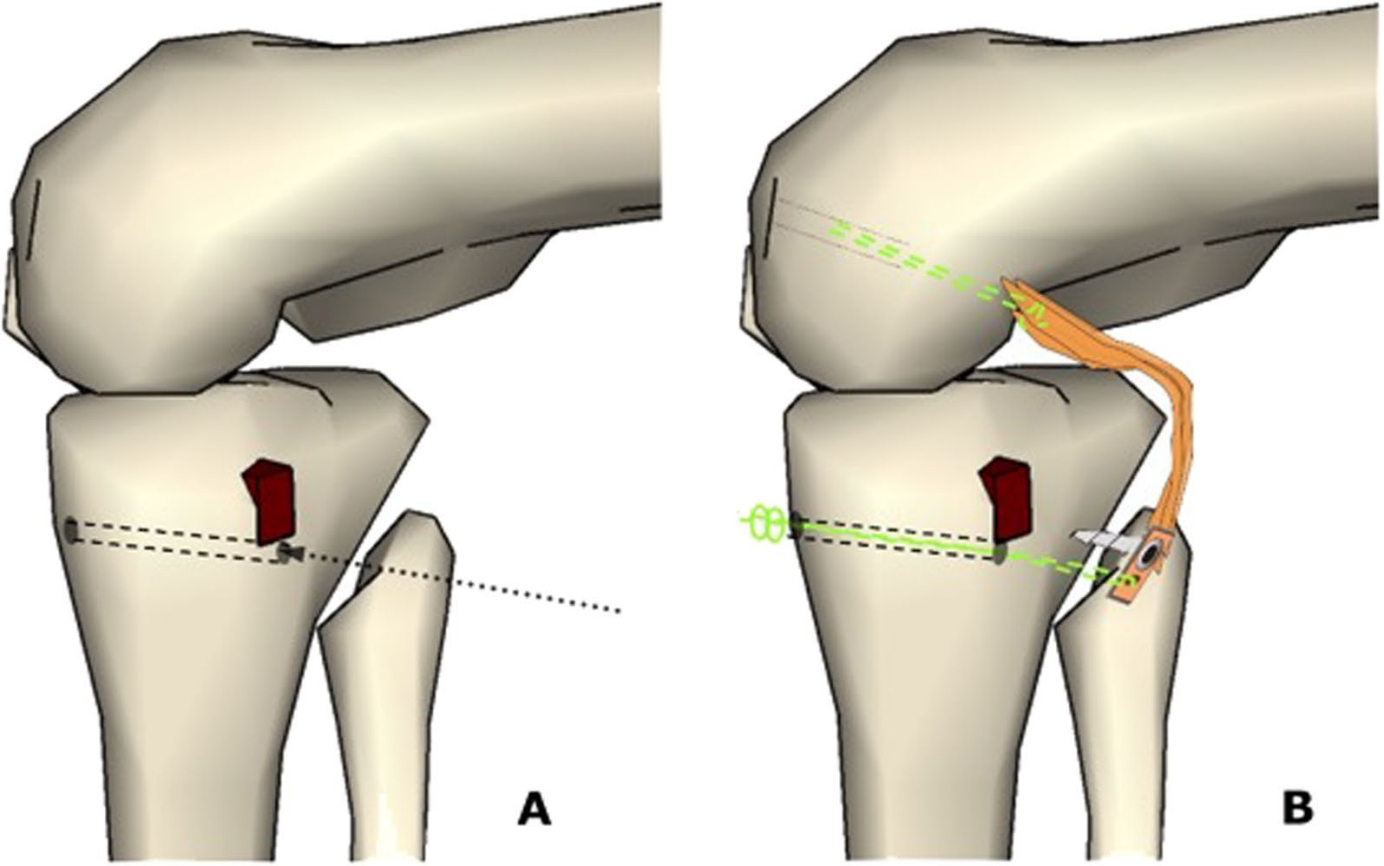
**A** Illustrates the 2.5 mm tunnel just below the PCL bone slot from the posterior to the anterior aspect of the knee joint, and **B** shows the passage of PCL graft controlled by Ethibond 5.0 thread in the transtibial and PCL femoral tunnels

**Fig. 9:**
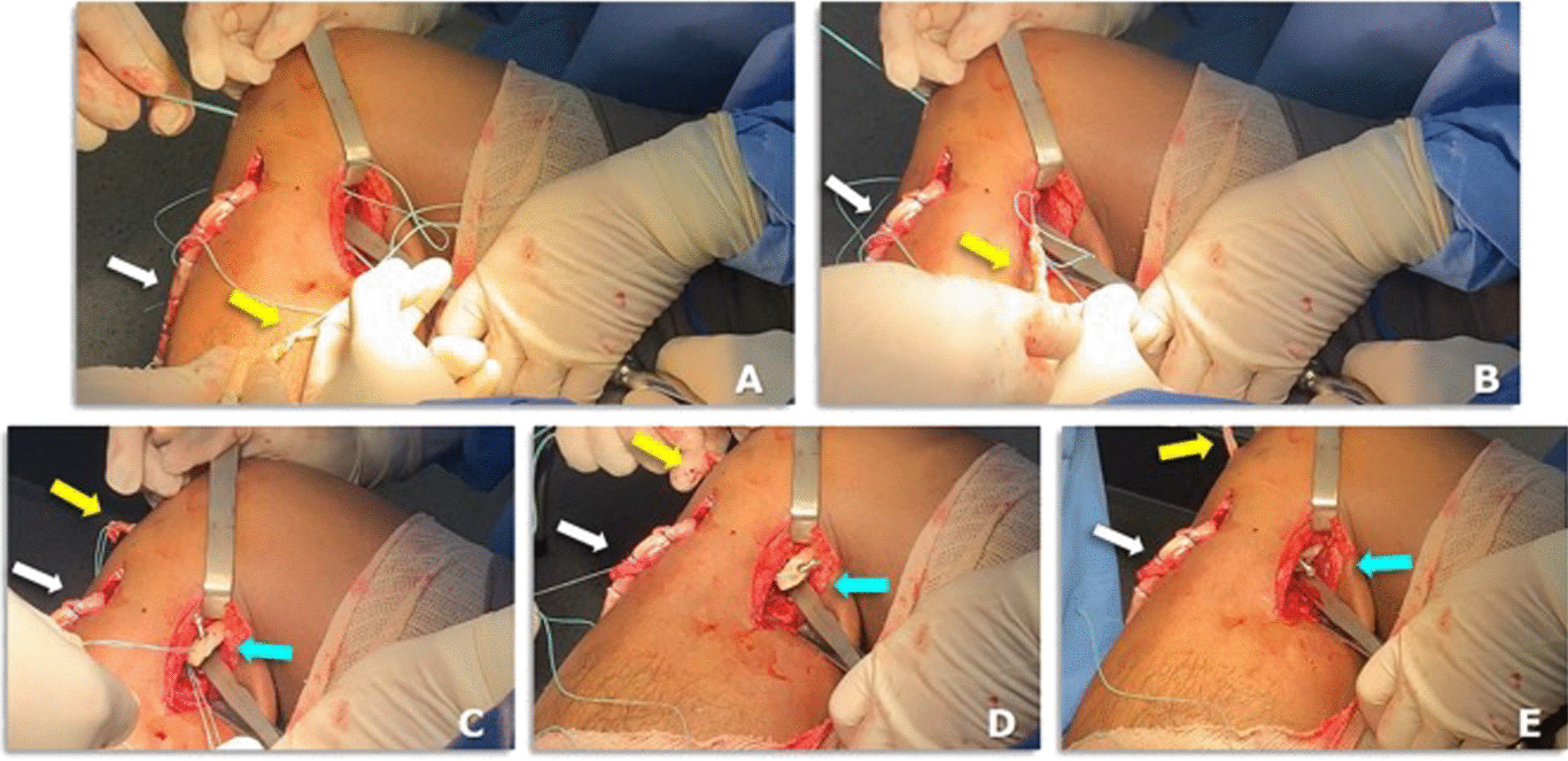
The PCL graft is shuttled into the knee joint through the tunnels using an Ethibond 5.0: **A**–**E** showing intraoperative views of the free end of PCL graft (yellow arrow), bone block with 3.5-mm cortical screw and washer of PCL graft (blue cyan arrow) and four stand of hamstring tendon of ACL graft (white arrow)
18. Then, the free end of the quadriceps tendon bone graft is passed from the posterior aspect into the knee joint up to the femoral tunnel using Ethibond thread 5.0, while the patellar bone block is inserted in the tibial slot guided guide a Ethibond thread 5.0 that was passed from tunnel drilled from the posterior to the anterior aspect of the tibia to tension it (Fig. 8B).
19. The patellar bone plug is positioned into the tibial slot using the PCL tibial inlay bone slot positioner, and further tension is applied using the Ethibond 5.0 suture to provide additional fixation (Fig. 8 B)
20. Tibial fixation starts by bone plug impaction using the PCL tibial INLAY bone slot IMPACTOR; 3.5mm cortical screw and washer fixation (24 or 46 mm); capsule suturing and pull-out fixation are performed (Fig. 10A–C).

**Fig. 10:**
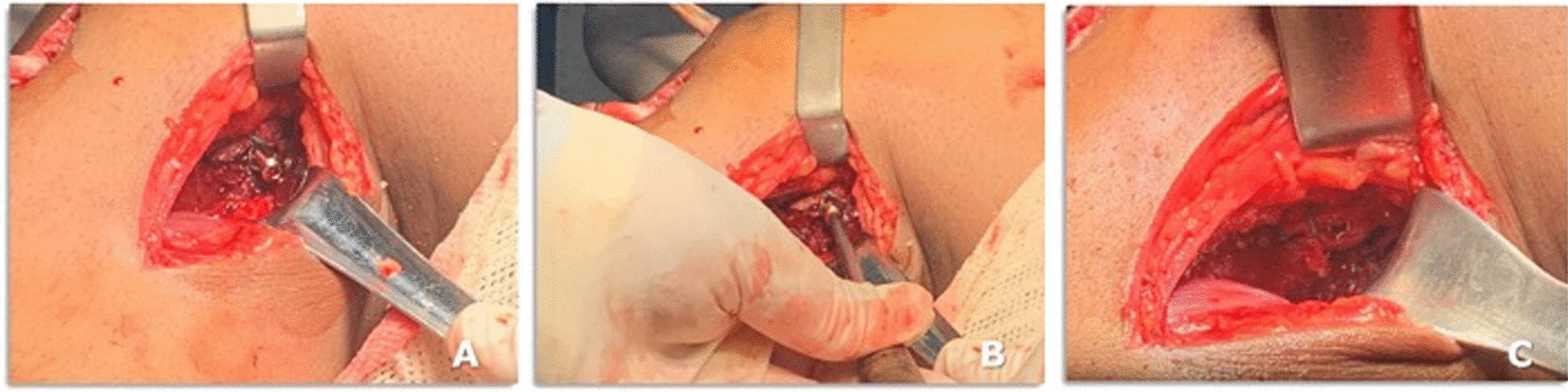
**A** PCL graft tensioning, impaction and fixation with a 3.5 mm cortical screw (24 or 26 mmm long) (**B**, **C** and **D**)
21. The ACL graft is then shuttled into the knee joint through the tunnels using an Ethibond 5.0 suture, and the knee is flexed and extended 20 times to tension the graft.
22. The ACL graft fixation starts on tibial side, using an interference screw 1 mm in diameter greater than the respective tunnel.
23. With the knee positioned at 90 degrees of flexion, the PCL graft is fixed on the femoral tunnel femoral fixation of PCL graft using an interference screw 1 mm in diameter greater than the respective tunnel, making sure that the knee is kept in neutral rotation. A gentle anterior drawer manoeuvre is performed at this stage to keep the knee anteriorly displaced for PCL femoral tunnel fixation, making sure that the normal contralateral knee is used as reference.
24. The ACL graft is fixed on the femoral side using an interference screw 1 mm in diameter greater than the respective tunnel (Fig. 11A and B)

**Fig. 11:**
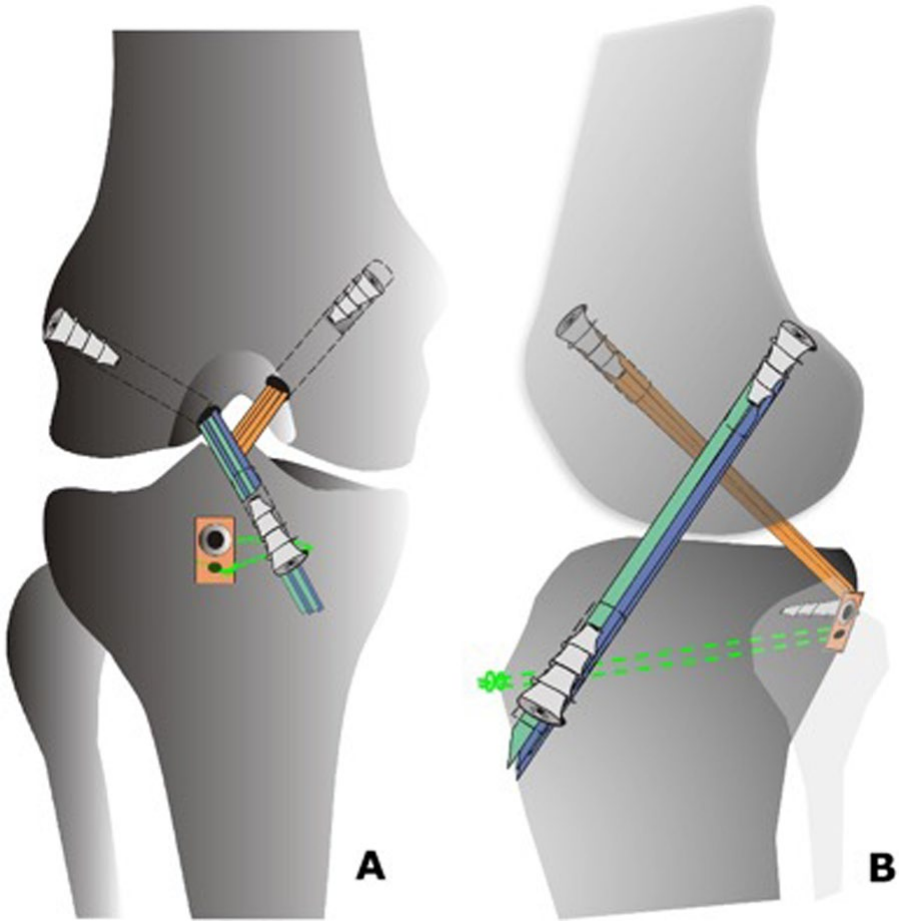
Drawing of bicruciate reconstruction after PCL and ACL fixation
25. Clinical manoeuvres are performed to ensure that posterior and anterior knee stability have been restored and that a full range of motion of the knee is possible.
26. The tourniquet is released, hemostasis is done, and the wound is sutured in a standard fashion
27. The knee is bandaged in a routine fashion and immobilized in a full extension brace

## Postoperative management

In the first two postoperative weeks, the knee is kept in full extension in a brace, and full weight-bearing is allowed keeping the knee immobilized in the brace. Using crutches is not mandatory, but patients feel more confident using them for the first 2 or 3 postoperative weeks. Cryotherapy and isometric exercises are introduced from the first postoperative day. The sutures are removed after two weeks. The brace is gradually unlocked after six weeks postoperatively, and motion is restricted to 45° in flexion until the six postoperative weeks.

After 12 weeks, the brace is completely removed, and rehabilitation focuses on progressively gaining full range of motion, which is expected to be achieved at around three months postoperatively.

## Key points of this surgical technique

### The knee posteromedial approach

- Removing the lateral knee holder facilitates positioning of the leg, and allows access to the posterior aspect of the knee with the patient supine
- The medial head of the gastrocnemius is retracted posteriorly and laterally so that the neurovascular structures were kept away from the surgical field

### The Piedade PCL tibial inlay set of instruments

Allows to approach the PCL tibial insertion site to produce a suitable tibial slot, of depth adequate to the dimensions of the graft bone plug.

### The central third of the quadriceps tendon graft should be

- At least 13 cm long, including the patellar bone plug (2 cm long, 1 cm wide and 1 cm thick).
- After harvesting the graft, two 2.5-mm drill holes are produced in the patella bone plug to be fixed by 3.5 mm cortical screw and washer on the PCL tibial bed.

### PCL graft fixation on the tibial side

- A 3.5-mm cortical screw is used in the proximal 2.5-mm tunnel in the patella bone plug, while in the distal 2.5-mm tunnel of in the patella bone plug a 5.0 Ethibond suture is used for pull-out fixation.
- The patella bone plug will be fixed by bone impaction (1), 5.0 Ethibond pull-out (2), a 3.5-mm cortical screw, and capsular suturing.

### Tunnel bone adjustment

- We prefer starting with a 6-mm bone tunnel just after arthroscopic assessment of intra-articular injuries and adjust the tunnel diameter after preparing the graft.

## Data Availability

The datasets generated during and/or analysed during the current study are available throughout the manuscript.

## Abbreviations

PCL: Posterior cruciate ligament
ACL: Anterior cruciate ligament
